# Primary percutaneous coronary intervention for cardio-cerebral infarction: a case report

**DOI:** 10.3389/fcvm.2023.1165735

**Published:** 2023-07-31

**Authors:** Tomomi Watanabe, Satoshi Kobara, Ryosuke Amisaki, Kazuhiro Yamamoto

**Affiliations:** Division of Cardiovascular Medicine and Endocrinology and Metabolism, Faculty of Medicine, Tottori University, Yonago, Japan

**Keywords:** acute myocardial infarction, acute ischemic stroke, coronary embolism, primary percutaneous coronary intervention, cardio-cerebral infarction, case report

## Abstract

**Background:**

Acute myocardial infarction (AMI) and acute ischemic stroke (AIS) are the leading causes of death globally. Cardio-cerebral infarction (CCI) is the rare occurrence of AMI and AIS, either simultaneously or one after the other. Treatment recommendations are not clear in case of the occurrence of AMI and AIS simultaneously, especially the strategy of primary percutaneous coronary intervention (PCI).

**Case presentation:**

We report consecutive seven case series of patients with CCI who underwent primary PCI in our institute. Comorbidities, strategy of primary PCI, and outcomes were investigated. All patients presented with the chief complaints associated with stroke. Atrial fibrillation (AF) was complicated in five of CCI patients, and four of AF patients were not anticoagulated. The major causes of stroke were cardiogenic and/or hemodynamic in this case series. All patients showed total occlusion in the culprit lesion, and six patients had other diseased vessels. Thrombus aspiration was mainly chosen as the reperfusion strategy in PCI. However, only two patients were diagnosed as definitive coronary embolism, and stenting was needed in six patients due to severe atherosclerotic lesion in culprit coronary artery. Final thrombolysis in myocardial infarction (TIMI) 3 flow was achieved only in four patients. Hemorrhagic complications occurred in three patients. Two patients died during in-hospital stay, and most had to be transferred for rehabilitation.

**Conclusions:**

CCI was a rare but fatal condition in patients who underwent primary PCI. Although CCI was associated with concomitant atrial fibrillation, organic coronary stenosis requiring stenting for revascularization was present in almost all the cases. Given the complexity of coronary artery lesions and high in-hospital mortality, further investigations are needed to determine the optimal treatment strategy.

## 1. Introduction

Acute myocardial infarction (AMI) and acute ischemic stroke (AIS) are the leading causes of death globally (1). In ST-segment elevation myocardial infarction (STEMI), primary percutaneous coronary intervention (PCI) contributes to high revascularization success rates, less cardiac events, and earlier discharge, and is effective even in patients with cardiogenic shock (2–4). In terms of AIS, thrombolysis has been a standard care, and endovascular thrombectomy has been used more aggressively in recent years, contributing to improved prognosis (5, 6) However, treatment recommendations are not clear in cases combining AMI and AIS. Cardio-cerebral infarction (CCI) is the rare occurrence of AMI and AIS, either at the same time (simultaneous or synchronous) or one after the other (metachronous) (7). It has been reported that the prognosis is poor with high rates of hemorrhagic events and in-hospital mortality, and primary PCI was performed in only 14% of cases of CCI (8). There were few reports on the treatment strategy of CCI, especially the strategy of primary PCI. Therefore, we retrospectively reviewed the records of 808 patients who underwent primary PCI for AMI from April 2012 to March 2022 in Tottori University Hospital. Patients with concurrent AIS during the same hospitalization were included in this case series, and obvious iatrogenic cerebral infarction (catheter induced) was excluded. Here, we report seven patients with CCI who underwent primary PCI in terms of the patient background, coronary angiographic characteristics, strategy of primary PCI, and clinical outcomes.

## 2. Case series

The ethics committee of Tottori University Hospital permits case reporting without obtaining written consent from each patient in accordance with the Ethical Guidelines for Epidemiological studies established by the Ministry of Health, Labour and Welfare, Japan.

The background of the seven patients is shown in Table 1. The background of the patients comprised age, gender, type of AMI, hypertension, smoking, diabetes, dyslipidemia, chronic kidney disease, history of myocardial infarction, taking aspirin, clopidogrel of prasugrel, oral anticoagulation, history of atrial fibrillation (AF), Killip classification, the Trial of Org 10172 in Acute Stroke Treatment (TOAST) classification (9), and CCI type. The type of CCI was classified based on the definitions reported by Habib et al. (10).

**Table 1:** The characteristics of the patients.

	Case 1	Case 2	Case 3	Case 4	Case 5	Case 6	Case 7
STEMI	+	−	+	+	+	+	+
HT	+	+	+	−	−	+	+
Smoking	−	−	−	−	+	−	−
Diabetes	−	−	−	−	−	−	−
Dyslipidemia	−	−	−	−	−	+	−
CKD	+	−	−	−	+	−	−
History of MI	−	−	−	−	−	−	−
ASA	−	−	−	−	−	+	−
Clopidogrel	−	−	+	−	−	−	−
Prasugrel	−	−	−	−	−	−	−
OAC	−	+	−	−	−	−	−
AF	+	+	−	+	−	+	+
Intracardiac thrombus	−	−	−	+	−	−	−
LVEF (%)	50	35	45	45	30	50	45
LA diam (mm)	41	51	29	48	41	26	37
CHADS 2	4	5	2	2	3	4	2
HAS—BLED	4	5	3	2	2	3	2
ARC—HBR	+	+	+	−	+	+	−
Killip	1	4	1	1	1	4	4
Infarct area in head CT	Rt. subcortical (watershed)	Rt. frontal (MCA area)	Bi. frontal (multiple)	Rt. parietal and Lt. temporal (multiple)	Lt. frontal/(MCA area)	Lt. frontal/(MCA area)	Lt. frontal/(MCA area)
MRA or CT angio findings	No significant stenosis	Not performed	Lt. ICA occlusion	Not performed	Not performed	Stenosis in Rt. MCA and PCA	No significant stenosis
TOAST	4	2	4	2	2	4	2
Arrival from AMI onset	Unknown	In hospital	6 h	2 h	4 h	1 h	10 h
Arrival from LKW	Unknown	6 h	6 h	In hospital	4 h	1 h	10 h
Interval between the onset of AIS and AMI	Unknown	7 days	0 h	Unknown	0 h	0 h	0 h
CCI type	1A	3	1A	2	1A	1A	1B

STEMI, ST-segment elevation myocardial infarction; HT, hypertension; CKD, chronic kidney disease; LKW, last known well; ASA, aspirin; OAC, oral anticoagulation; AF, atrial fibrillation; LVEF, left ventricular ejection fraction; LA diam, left atrial diameter; ARC-HBR, academic research consortium—high bleeding risk; CT, computed tomography; MRA, magnetic resonance angiography; MCA, middle cerebral artery; ICA, internal carotid artery; PCA, posterior cerebral artery; TOAST, Trial of Org 10172 in Acute Stroke Treatment Classification; AMI, acute myocardial infarction; AIS, acute ischemic stroke; CCI, cardio-cerebral infarction.

Patients ranging in age from 40s to 90s were enrolled in this case series. Four patients were female and have fewer common coronary risk factors. Most patients had high bleeding and embolic risk. AF was highly consistent with CCI patients, and four of five AF patients were not anticoagulated. The cases with untreated AF consisted of elderly patients who had no cerebrovascular events and did not choose anticoagulation before admission. All patients presented with the chief complaints associated with stroke: hemiplegia, dysarthria, ataxia, apraxia, loss of speech, loss of consciousness, and hemispatial neglect on admission. The major causes of stroke were cardiogenic and/or hemodynamic in this case series.

No patient underwent endovascular treatment or thrombolytic therapy for AIS in this case series, because most was unstable or had a long or uncertain time since last known well. Because of unstable condition, adequate neurological assessment with cerebrovascular imaging such as contrast-enhanced computed tomography (CT) or magnetic resonance angiography was not conducted in three cases.

The patients were pretreated with 200 mg aspirin and 300 mg clopidogrel or 20 mg prasugrel as the loading doses, followed by intravenous heparin boluses during the procedure to maintain an activated clotting time of >250 s. Before PCI, coronary angiography was performed, and the thrombolysis in myocardial infarction (TIMI) flow and Rentrop grade were evaluated at the initial angiography. The PCI strategy such as thrombus aspiration, plain old balloon angiography (POBA), and stenting was left to the discretion of each physician. Glycoprotein IIb/IIIa inhibitors are not available in Japan.

The angiographic and therapeutic characteristics are shown in Table 2. A diseased vessel was defined as numbers of non-culprit vessel that showed over 75% stenosis. Coronary embolism was judged as definite or probable by using the diagnostic criteria previously reported (11).

**Table 2:** Angiographic and therapeutic characteristics.

	Case 1	Case 2	Case 3	Case 4	Case 5	Case 6	Case 7
Culprit vessel	RCA	LAD	LAD	RCA	RCA	RCA	LAD
Diseased vessel	3	2	2	1	2	2	2
1^st^ TIMI	0	0	0	0	0	0	0
Collateral	3	1	0	1	1	1	3
Calc	+	+	−	−	−	−	+
Emboli	No	Probable	No	Definite	Definite	Probable	Probable
Aspiration	+	+	−	+	+	−	+
Aspirated thrombus	None	None		Red thrombus	Red thrombus		None
POBA	+	+	+	−	+	+	+
Stent	+	+	+	−	+	+	+
Post-dilatation	+	+	+	−	+	−	+
Stent diameter	3, 2.5	2.75	3	n	3	3	2.5
Stent length	28 + 23	26	12	n	23	26	30

RCA, right coronary artery; LAD, left anterior descending; calc, calcification; POBA, plain old balloon angioplasty.

All the patients showed total occlusion in the culprit lesion, and six patients had other diseased vessels. Only two patients were diagnosed as definitive coronary embolism. Thrombus aspiration was mainly chosen as the reperfusion strategy in PCI. However, retrieved visible thrombus was obtained in only two cases. Stenting was needed in six patients due to severe atherosclerotic lesion in culprit coronary artery, and relatively long stenting and post-dilation also tended to be required. Although one case (case 5) was diagnosed as a definitive coronary artery embolism based on the algorithm described above (11), the patient underwent stenting due to the concomitant atherosclerotic lesion.

Treatments post-PCI and outcomes are shown in Table 3. IABP was used in three patients, but final TIMI 3 flow was achieved in four patients (57.1%). Three patients required intensive antithrombotic therapy including dual antiplatelet and anticoagulation, and hemorrhagic complications occurred in three patients. Despite AF, case 2 was not prescribed anticoagulation therapy because of her advanced age. Conversely, AF was not detected in case 3, but he underwent triple therapy (dual antiplatelet therapy and anticoagulation) because the embolic cause could not be ruled out. In-hospital mortality was 28.6%, and four of five survived patients were transferred to satellite hospitals.

**Table 3:** Treatments after PCI and outcomes.

	Case 1	Case 2	Case 3	Case 4	Case 5	Case 6	Case 7
Final TIMI	3	3	3	2	2	2	3
MCS	−	IABP	IABP	−	−	IABP	−
ASA	+	+	+	−	+	+	−
Clopidogrel	+	+	+	−	+	+	+
Prasugrel	−	−	−	−	−	−	+
OAC	+	−	+	+	−	+	+
Hemorrhagic stroke	−	+	−	+	−	−	−
Bleeding	−	+	−	+	−	+	−
Re-stroke	+	+	−	−	−	−	−
Re-MI	−	−	−	−	−	−	−
In-hospital death	+	+	−	−	−	−	−
Cause of death	Pneumonia	Heart failure					
Transfer			−	+	+	+	+
Initial BI	0	0	10	10	10	15	0
BI			75	85	45	50	30

TIMI, thrombolysis in myocardial infarction trial flow; MCS, mechanical circulatory support; ASA, aspirin; OAC, oral anticoagulation; MI, myocardial infarction; BI, Barthel index; IABP, intra-aortic balloon pumping.

**Figure 1:**
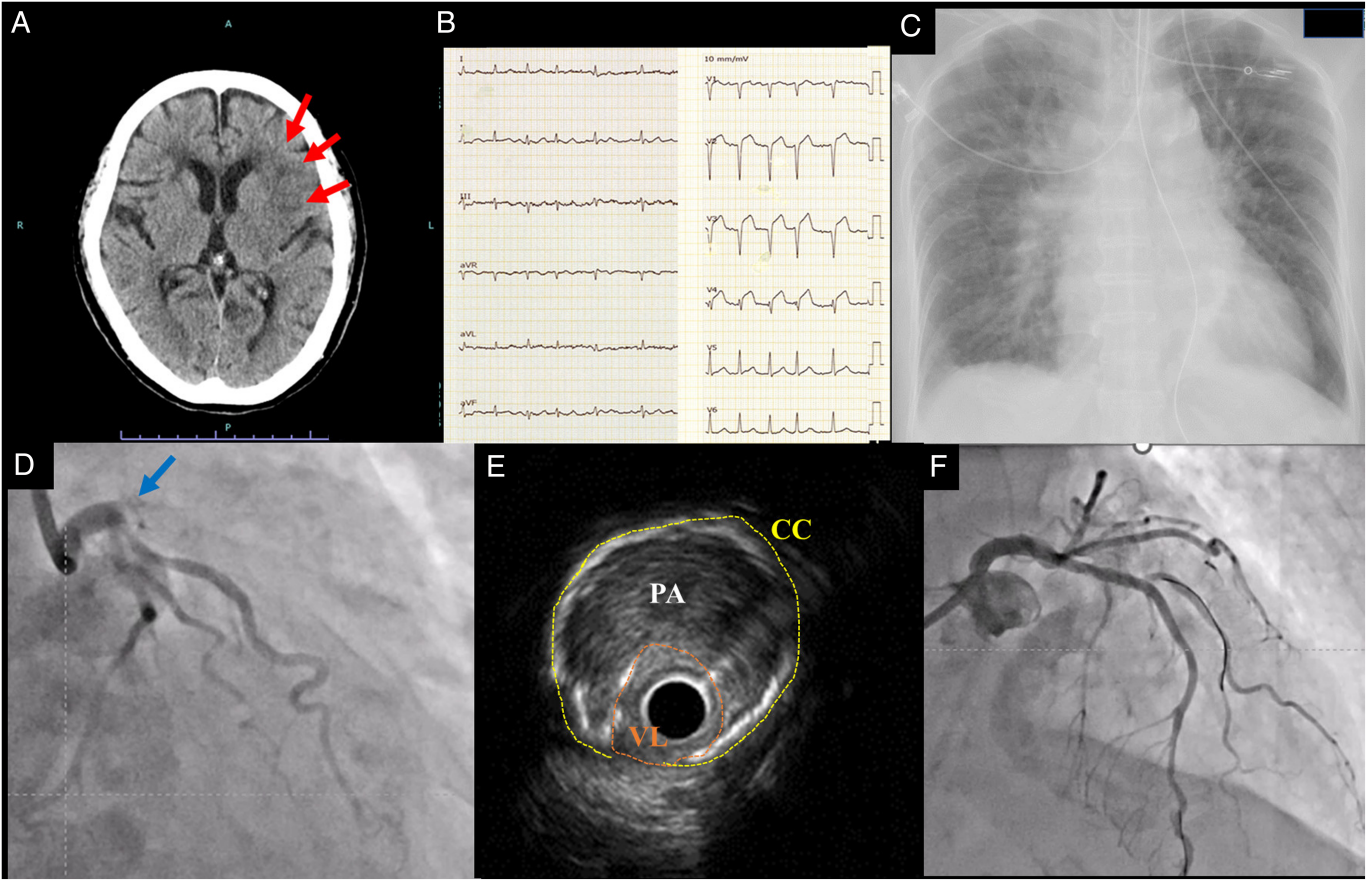
Representative case of cardio-cerebral infarction requiring coronary stenting for myocardial infarction. Head CT showed low density area in left middle cerebral artery area (red allows) at admission (**A**). Electro-cardiogram showed ST-segment elevation in anterior leads (**B**). Chest X ray showed pulmonary congestion (**C**). Coronary angiography showed total occlusion at proximal of left anterior descending artery (blue arrow) (**D**). Intravascular ultrasound showed that vessel lumen (VL) was narrowed (highlighted in orange) by plaque area (PA) and circumferential calcification (CC) (highlighted in yellow) due to atherosclerotic changes in the culprit lesion (**E**). TIMI 3 flow was obtained after stenting, and the stented lesion was indicated (**F**).

A representative case of CCI requiring stenting for myocardial infarction due to severe atherosclerotic lesions (case 7) is shown in Figure 1. The patient was admitted to the emergency room with a chief complaint of impaired consciousness and right hemiplegia for the past 9 h. She was diagnosed with AIS in the left middle cerebral artery area, and obvious ischemic changes were already detected by head CT (Figure 1A). However, the electro-cardiogram showed AF and ST-segment elevation in the anterior leads (Figure 1B). The chest x-ray showed cardiomegaly and pulmonary congestion (Figure 1C). Primary PCI was performed because of suspected pulmonary edema due to anterior STEMI. Angiography showed total occlusion in the proximal of left anterior descending artery (Figure 1D). Although we strongly suspected coronary embolism because of the concomitant AF and AIS, repeated thrombectomy and balloon angioplasty were ineffective. Intra-vascular ultrasound showed severe calcification and diffuse atherosclerotic lesion in the proximal to the middle of left anterior descending artery (Figure 1E). Stenting was effective to gain the recanalization, and post-dilatation was performed. Anticoagulation and antiplatelet therapy were administered to prevent recurrence, and she was transferred to a satellite hospital 2 months later without hemorrhagic stroke (Figure 1F).

## 3. Discussion

We described seven patients with CCI who underwent primary PCI. AF was highly complicated in CCI patients (71.4%), and thrombus aspiration was mainly selected as the initial reperfusion strategy in PCI. However, stenting was needed in six of seven patients due to severe atherosclerotic lesion in culprit coronary artery. In-hospital mortality was as high as 28.6%, and most of the survived patients required transfer to satellite hospitals.

Considering the current case series, the prevalence of CCI was 0.87% in AMI patients who underwent primary PCI. The prevalence of diabetes was lower and that of AF was higher in CCI patients as compared with typical ACS patients in Japan (12). In this report, the CCI patients were mostly without the prescription of anticoagulant before the admission and were clinically treated as Type 2 (cardio-embolic) of the TOAST classification. However, coronary lesions resisted stentless strategy including aspiration, and finally required stenting. Yeo et al. (13) also reported that three of three CCI patients who underwent PCI required stenting.

The use of PCI with stenting requires peri-procedural heparin infusion, and long-term use of dual antiplatelet therapy. Especially, stenting in AMI patients requires more intense antiplatelet therapy, including loading dose. Previous report suggested that this was the reason for hesitation regarding primary PCI in acute stroke patients, but two of two CCI patients without primary PCI died (14). A small retrospective study by Schmidbauer et al. (15) showed that the incidence of intracerebral hemorrhage in AIS patients was not increased by cardiac catheterization including PCI. Coronary stenting should not be necessarily hesitated in cases of STEMI or obvious hemodynamic instability even in the acute phase of CCI.

Post-PCI antithrombotic therapy should be considered integrated as appropriate antithrombotic therapy with the target of reducing embolic events and bleeding complications in the current guidelines (16). In this case series, three patients were treated with intensive antithrombotic therapy including dual antiplatelet and anticoagulation therapy. This may be due to the inclusion of cases before the current guidelines were disseminated. If treatment is based on this current strategy, de-escalation of antithrombotic therapy may reduce bleeding complications even if stenting is used.

Ideally, comprehensive examinations and simultaneous treatment for infarction of the two organs should be performed. Although intravenous thrombolysis for AIS and AMI seems to be an appropriate approach in this context, there is a dilemma regarding the different indications and dosages of intravenous alteplase in the treatment of AIS and AMI (14). Furthermore, thrombolysis does not always provide a recanalization, and endovascular treatments are increasingly used in AIS (17). Treatment algorithms and concepts including endovascular treatment have been proposed, and further research is needed for the ideal management strategy that provides the best outcomes (13, 18). This case series reported that AMI was mostly attributed to severe arteriosclerotic coronary artery disease rather than to an embolic mechanism in patients with CCI. It suggests that stenting is likely to be necessary in the treatment of CCI even if there is a concomitant embolic event.

CCI is a rare condition in patients who underwent primary PCI. Although CCI was frequently associated with concomitant AF, organic coronary stenosis requiring stenting for revascularization was present in most cases in this study. Given the complexity of coronary artery lesions and high in-hospital mortality, further investigations are needed to determine the optimal treatment strategy.

## Data Availability

The original contributions presented in the study are included in the article, further inquiries can be directed to the corresponding author.
